# Internet-enabled pulmonary rehabilitation and diabetes education in group settings at home: a preliminary study of patient acceptability

**DOI:** 10.1186/1472-6947-13-33

**Published:** 2013-03-05

**Authors:** Tatjana M Burkow, Lars K Vognild, Geir Østengen, Elin Johnsen, Marijke Jongsma Risberg, Astrid Bratvold, Tord Hagen, Morten Brattvoll, Trine Krogstad, Audhild Hjalmarsen

**Affiliations:** 1 Norwegian Centre for Integrated Care and Telemedicine, University Hospital of North Norway, P.O. Box 35, Tromsø, N-9038, Norway; 2 Norut, P.O. Box 6434, Forskningsparken, Tromsø, N-9294, Norway; 3 Hendig AS, Granlia 23, Fetsund, N-1900, Norway; 4 Department of Pulmonary Rehab, Heart and Lung Clinic, University Hospital of North Norway, P.O. Box 100, Tromsø, N-9291, Norway; 5 Medical Clinic, University Hospital of North Norway, Tromsø, Norway; 6 The University's Centre for Flexible Education, University of Tromsø, Tromsø, N-9037, Norway; 7 Norsk Helsenett SF, Tromsø, Norway

**Keywords:** Internet, Home-based, Group-based, Pulmonary rehabilitation, Diabetes self-management education, COPD, Diabetes, Chronic diseases, Web technology, Open source software

## Abstract

**Background:**

The prevalence of major chronic illnesses, such as chronic obstructive pulmonary disease (COPD) and diabetes, is increasing. Pulmonary rehabilitation and diabetes self-management education are important in the management of COPD and diabetes respectively. However, not everyone can participate in the programmes offered at a hospital or other central locations, for reasons such as travel and transport. Internet-enabled home-based programmes have the potential to overcome these barriers.

This study aims to assess patient acceptability of the delivery form and components of Internet-enabled programmes based on home groups for comprehensive pulmonary rehabilitation and for diabetes self-management education.

**Methods:**

We have developed Internet-enabled home programmes for comprehensive pulmonary rehabilitation and for diabetes self-management education that include group education, group exercising (COPD only), individual consultations, educational videos and a digital health diary. Our prototype technology platform makes use of each user’s own TV at home, connected to a computer, and a remote control. We conducted a six-week home trial with 10 participants: one group with COPD and one with diabetes. The participants were interviewed using semi-structured interviews.

**Results:**

Both home-based programmes were well accepted by the participants. The group setting at home made it possible to share experiences and to learn from questions raised by others, as in conventional group education. In the sessions, interaction and discussion worked well, despite the structure needed for turn taking. The thematic educational videos were well accepted although they were up to 40 minutes long and their quality was below TV broadcasting standards. Taking part in group exercising at home under the guidance of a physiotherapist was also well accepted by the participants. Participants in the COPD group appreciated the social aspect of group education sessions and of exercising together, each in their own home. The digital health diary was used as background information in the individual consultations and by some participants as a self-management tool. Participant retention was high, with no dropouts. None of the participants reported that the six-week duration of the home programmes was too long.

**Conclusions:**

The Internet-enabled programmes for home-based groups in pulmonary rehabilitation and diabetes education were generally well accepted by the participants. Our findings indicate that conventional programmes have the potential to be delivered in socially supportive group settings at home.

## Background

Chronic diseases cause a huge burden to society, and increasing numbers of people are suffering from major chronic diseases such as chronic obstructive pulmonary disease (COPD) and diabetes. The World Health Organization (WHO) estimates that 65 million people have COPD and that by 2030 COPD will be the third leading cause of death globally [1]. Diabetes is even more common; WHO estimates that there are 346 million people with the disease worldwide, some 3.4 million died from the disease in 2004, and it is expected that the number of deaths will double by 2030 [2].

Pulmonary rehabilitation is recognised as a standard and important part of COPD management [3-6]. It is a comprehensive, multidisciplinary intervention; a pulmonary rehabilitation programme includes education, training, psychosocial support, and patient assessment [3]. Outcomes include improved health-related quality of life, reduced dyspnoea and increased exercise capacity [5]. Pulmonary rehabilitation is commonly offered as hospital- or community-based outpatient programmes in group settings, lasting 8 to 12 weeks.

Diabetes self-management education is an important part of diabetes care for improved patient outcomes [7]. Diabetes education is often organised in group settings, due to the increasing number of people with diabetes and the pressure on health staff resources [8]. Outcomes include reduced fasting blood glucose levels, reduced glycated haemoglobin (HbA1c), improved diabetes knowledge, and reduced need for medication [8].

For people living in rural areas, often with long travelling distances, it can be difficult, burdensome or even impossible to participate in the conventional centre-based education and rehabilitation programmes. For pulmonary rehabilitation, travel and transport are barriers to both uptake and completion [9]. Home-based programmes have the potential to overcome these barriers, and to improve access to education and rehabilitation in underserved areas. However, the possible lack of opportunity for group support may be a potential drawback of home-based pulmonary rehabilitation [3].

We have developed home programmes with patient groups for multidisciplinary and comprehensive pulmonary rehabilitation and diabetes self-management education. The home programmes are based on existing outpatient programmes. Our prototype Internet-connected technology platform enables the use of multiparty videoconferencing, a health diary, health-related educational materials, and vital sign monitoring [10,11]. As the user interface at home, we use familiar technologies: the user’s own TV and a remote control.

Related work on home-group-based comprehensive pulmonary rehabilitation is limited. Home-based pulmonary rehabilitation has generally target exercise training in daily life, and everyday physical activity for individual patients, without group support. However, studies have been conducted on live group exercising at home [12,13]. For diabetes, videoconferencing has been used for individual case management at home [14]. We are not aware of any studies where videoconferencing is used for home-groupbased diabetes education.

Important factors in the development of health technologies and services are acceptability to patients (the extent to which services are generally approved or used, the perceived usefulness of services, and patients’ satisfaction) as well as the perceived usability of the technology.

The usability results from the trial have been published previously [10,11]. The findings were that the technology was easy to use regardless of patients' computer skills, with little time and training required to learn.

In this paper, we assess patient acceptability of the delivery form and the programme components.

## Method

We performed a trial with Internet-enabled programmes based on home groups for comprehensive pulmonary rehabilitation and diabetes self-management education. The purpose of this feasibility study was to assess patient acceptability of the delivery form and the components of the home programmes.

### Pulmonary rehabilitation programme at home

The home-based pulmonary rehabilitation programme we developed was based on an existing comprehensive and multidisciplinary outpatient programme at the University Hospital of North Norway. The outpatient programme encompassed supervised group-based exercise training, individual exercising, group education and patient outcome assessment. The programme lasted eight weeks, the participants enrolled at the same time, and they participated twice a week at the rehabilitation centre.

The home programme was tailored to people with very severe COPD, GOLD stage IV, receiving long-term oxygen therapy (LTOT), due to their need for adequate patient education [15]. The burden of oxygen therapy itself has also been found to be a factor associated with non-attendance at pulmonary rehabilitation [16]. The home-based rehabilitation programme included weekly group education, group exercising, and individual consultations, lasting six weeks.

A multidisciplinary team of health professionals including a pulmonary nurse, physiotherapist, specialist and nutritionist delivered the group education. The education sessions focused on general themes in COPD as well as themes specific to LTOT, such as oxygen use while travelling. The healthcare personnel had the role of mediators in the education sessions. In preparation for each session, the participants were requested to watch one or more tailored educational videos we made on the subject. The videos, featuring the healthcare personnel delivering the education sessions, were not of professional TV broadcasting quality, and varied in length from 10 to 40 minutes.

A physiotherapist supervised the group exercise sessions, and a video featuring the same physiotherapist was intended for additional exercising at home during the week. The home exercise programme was based on the exercise programme at the rehabilitation centre, and was intended to strengthen upper and lower extremities and to increase thorax flexibility. Background music was used, and the physiotherapist guided and monitored the group exercise sessions based on visual feedback. The exercise sessions lasted half an hour.

The pulmonary nurse and physiotherapist alternated between giving the individual consultations, which were intended for elaboration upon individual health issues that were not suitable to discuss in the presence of peers. A digital health diary on TV, to be updated daily, was intended both as background information for the consultations and for self-management purposes. The digital health diary included questions on oxygen usage, medication, oxygen saturation, physical activity, well-being, and nutrition.

As part of the programme, an in-person start-up meeting was arranged. Our assumption was that the participants would be more comfortable as a videoconferencing group if they had first met in person. The meeting included a supervised group exercise session, a LTOT specific lecture, and a presentation and demonstration of the technology.

For practical and financial reasons, the home programme was two weeks shorter than the outpatient rehabilitation programme. We considered the duration adequate to provide insight into patient acceptability.

### Diabetes group education at home

The home education programme for diabetes self-management was developed based on two existing programmes at the University Hospital of North Norway: 1) A group-based outpatient programme in diabetes self-management education given by a multidisciplinary team of health professionals over two consecutive days and 2) a phone support line for individual consultations run by the diabetes nurse coordinating the outpatient programme.

The diabetes home programme included weekly group education and individual consultations. We wanted to assess patient acceptance of a home-group education programme lasting several weeks, and six weeks was chosen for practical and financial reasons.

A multidisciplinary team of health professionals including a diabetes nurse, a nutritionist and a physiotherapist provided the group education sessions, where they also had the role of mediators. In preparation for each session, the participants were expected to watch one or more tailored educational videos we had made. As for the COPD programme, the videos were not of professional TV broadcasting quality, they varied in length from 10 to 40 minutes, and they featured the same healthcare personnel who delivered the education sessions.

There were weekly individual consultations with the diabetes nurse. Each participant had a digital health diary on TV. As for the COPD programme, the diabetes diary was to be updated daily, for self-management purposes and to provide background information for the individual consultations. It included blood glucose readings and questions on physical activity, well-being and nutrition.

For this group, too, there was an in-person start-up meeting so that the participants could get to know each other before the videoconferencing group sessions. The in-person start-up meeting included lectures, and a presentation and demonstration of the technology.

### The technical platform

The prototype technical platform consisted of the user’s own TV connected to a computer, the Residential Patient Device, and a remote control, dedicated to this purpose [10,11]. We used a small form-factor computer (Soltek Qbic) running Slackware Linux as the Residential Patient Device, and implemented the software system using web technologies and open-source software components. The Residential Patient Device provides local data storage, a secure and reliable communication channel over the Internet between the home and the public health service, and functionality for orchestrating comprehensive home-based health services. This includes the possibility of text- and video-based educational material, multiparty videoconferencing, and a health diary with disease-related questions and wireless transmission or manual entry of sensor data (in our case oxygen saturation, heartbeat and blood glucose values). The questions in the diary were multiple-choice with predefined answers.

For the audiovisual communication, a web camera and a wired headset with a microphone were used. The health diary information was transferred to the hospital information system in advance of the weekly individual consultation. The patient did this by explicitly pressing a ‘send’ button. Automatic transfer of the health diary information would also have been possible; however, from a self-management perspective, we decided to give the user explicit control of the health diary.

The healthcare professionals were located at the hospital outpatient clinic. A commercial stand-alone videoconferencing system (Tandberg Maestro) was used in the group sessions, with a large image projected onto the wall. A computer-based open-source videoconferencing system (GnomeMeeting) was used for the individual consultations.

With the commercial stand-alone videoconferencing system, six sites could be connected simultaneously. In the group sessions, the participants could see each other on the TV screen. The screen was divided into one large and five smaller images. In the education sessions, the largest image was that of the person speaking, and the other participants were visible in the smaller images. In the exercise sessions, the physiotherapist was always allocated the largest image. During exercising, the loudspeaker on TV was used for accompanying music, and the participants removed their headset in order to avoid breathing noise.

The users’ broadband Internet connections had an upload bandwidth of 250–400 kbps, and downlink bandwidth of 1000–2000 kbps. This was the fastest available to the participants at that time. Information security measures in the system included encryption (storage and transmission), authentication and automatic logout after a period of non-usage.

### The participants

Five participants with COPD in GOLD stage IV receiving LTOT were recruited; two were female and three male. All were over 40, which is the most common age range in which people are diagnosed with COPD [17]*.* One participant was in the age group 45–54, two were in the age group 55–64, and two were in the age group 65–74. They all lived in the county of Troms (an area of 25,000 square km with a scattered population). For three participants, the distance between home and the outpatient clinic was in the range 0.5–9 km, and for two participants it was in the range 80–90 km. The participants had previously participated in pulmonary rehabilitation, three of them at the hospital outpatient clinic. Four were living alone, and one with family. All were retired. Two of the participants were non-computer users, two used the Internet at least once a week but not every day, and one used the Internet daily or nearly every day.

Five participants with diabetes were recruited, three female and two male. One of them was in the age group 35–44, two were in the age group 45–54, one was in the age group 55–64 and one in the age group 65–74. None had attended an education programme for diabetes self-management previously. However, several had used other sources for information on diabetes. One of the participants was living alone, and the other four with family. Three of the participants were retired. They all lived in the county of Troms. The distance from the outpatient clinic was around 5 km for two of them; for the other three it was in the range 90–160 km. One of the participants was a non-computer user, two used the Internet at least once a week but not every day, and two used the Internet daily or nearly every day.

We considered a group size of five adequate to provide insight into acceptability, even though this was a limitation imposed by the specific videoconferencing technology.

Healthcare personnel at the hospital outpatient clinic recruited the participants. The participants received written and oral information about the project, and gave written informed consent for the study.

### Ethical and legal issues

An application for approval was sent to the regional ethics committee. The committee decided that ethical approval was not needed for the study. To fulfil the requirements of Norwegian legislation on processing of personal data, risk assessment was performed.

### Training and technical support

Both participant groups were given a demonstration of the system at the in-person start-up meeting, and had an additional technical walk-through when the system was installed at their homes. They also had a short user manual, and could call technical support during the trial period. A videoconferencing test session was held with each of the patient groups before the first group session. This served both as a technical test at all sites, and as an opportunity for the participants to become more familiar with the technology.

The healthcare staff participating in the trial were offered an introductory session, lasting approximately an hour, to familiarise themselves with the videoconferencing system. During the videoconferencing sessions, technical support staff were available to provide help if needed. All the healthcare professionals had extensive experience of providing hospital-based outpatient pulmonary rehabilitation and diabetes education. One of them had extensive experience of using videoconferencing, while the others had little or no experience. Both genders were represented.

### Data collection and analysis

We developed and used a semi-structured interview guide common to both diabetes and COPD, with additional questions on exercising for the COPD participants. The interview guide contained principal open-ended questions developed to assess patient acceptability of the home programme delivery and of the different programme components. The primary themes were the general impression of the home programme and user perceptions of the in-person start-up meeting, the education sessions, the educational videos, the exercising sessions and the exercise video (COPD only), the individual consultations and the digital health diary.

The participants were interviewed individually by one of the authors, face to face at home for approximately one hour, after the trials in late 2005. The interviews were taped and transcribed. The interview quotes in this article were translated into English from the informants’ native language, Norwegian. They are labelled P1-P5 for the COPD participants and P6-P10 for the diabetes participants.

Three of the authors read through and structured the transcribed material, and an additional two authors participated in the further analysis. Structuring was performed by means of a “meaning categorization” [18], in which the material was categorised according to the study's main themes and relevant sub-categories. This structuring functioned as a sorting and preparation of the material before extraction of positive and negative aspects regarding the issues raised and responded to by the participants. The coded material was organised in a matrix to provide an overview of the categories and make it easier to study the relationships between them [19]. Thus an “issue-focused” analysis was performed [20], aimed at compiling and comparing the respective participants' responses, a so-called cross-case analysis [21].

## Results

The results from the interviews with the COPD and diabetes participants are presented together, and where important differences were encountered, they are specifically mentioned.

### General impression

The participants were generally very positive about the home-based programmes. This was illustrated in statements such as*:*

Positive, the whole thing, this is our future. (P6)

Why on earth has nobody thought of this before! (P3)

All in all, I would say it was positive. (P4)

A good way of doing it. (P10)

### Group education

Receiving education at home was well accepted by the participants. Both the education in itself and the opportunity to learn from peers were emphasised:

Yes, it was very interesting. Then you can hear about how other people experience a situation similar to the one you are in yourself. (P7)

Many issues cropped up from the other people who took part. (P1)

You can all talk and you can listen to each other … some people have this to say, others may have that to say. (P8)

What I thought about it was, wow, if I could have learned this straight away when I found out that I had become diabetic, it would have been so much easier then. (P9)

The communication consisted mainly of dialogue in the form of questions and answers to the lecturer, with less discussion directly between the participants. Everyone had a chance to speak, but the technique imposed some limitations:

You could get through when you wanted and say what you thought. (P5)

…several people started to talk at the same time. And then you stopped again when you heard other people speaking, and then everyone started talking at the same time again. (P1)

The educational videos were well received by the participants; they found the content relevant and that the videos gave peace and quiet for learning. They also found the duration appropriate and the lack of broadcast TV quality not a problem. The opportunity to ask questions in the TV sessions to clarify issues after watching the video was also perceived as positive:

First we watch the video … then the lecturer talks about the video, and then you can ask questions to get things clear. (P7)

All participants had watched the educational videos before the group sessions.

### The in-personstart-up meeting

Four participants with COPD and three with diabetes attended the in-person start-up meeting held for their group. The other three could not attend due to practical issues or illness.

Those who had attended were positive to a start-up meeting, but those unable to attend also felt that the home programme worked well, as expressed by one of them:

It was actually interesting to get to know them, the way we did. (P8)

### Supervised group exercise training and the exercise video

Only the COPD programme contained exercise-training sessions. The physiotherapist-supervised exercise sessions were well received by the participants, one of whom said:

Actually it went almost as well as it did over there [at the rehabilitation centre] … It was the same feeling. You would think that you would get absorbed in looking at the others, but in fact you didn't. You concentrated on [the physiotherapist] the whole time. (P5)

Another participant emphasised the social aspect of exercising together, even though it was on TV:

And to exercise together with other people, never mind that it is happening at home in their own homes, it means more than you might think. Because there's the social aspect as well. Because even though it's happening on TV you don't think about that. It's just as though we were together with each other. (P3)

Some participants thought there should have been more group exercise sessions, and three of the five participants exercised using the follow-along exercise video.

### The social aspect

We did not ask about the social aspect; it emerged spontaneously in some of the interviews with the COPD participants. As expressed by two of them:

The social aspect, I heard several mention that. (P5)

It was just as though we hadn't done anything other than sitting at home in our own living room and chatting with each other via the TV. So, that way you also included the social bit, which is hugely important. (P3)

However, one COPD participant commented on a lack of eye contact in the video sessions compared to meeting people in person:

I like to look people in the eye when I talk to them … it was not quite the same as having them sitting over there on the sofa or in a chair in front of me. (P2)

### The individual consultations and the health diary

The individual consultations were well perceived by both patient groups. They provided an opportunity to discuss personal issues, and they were seen almost as face-to-face conversations by some participants:

It was almost like sitting and talking face to face. (P9)

We could ask about things that you might not want to ask in public. (P2)

However, the need to ask questions in the individual consultations varied among the participants, for example, depending on the stability of the disease. The health diary was used in the individual consultations, and a few participants used the health diary in a self-management perspective:

If I had not exercised on one day, that was bad (laughter). If you had done a little bit, that was OK, as long as the blood sugar stayed stable. (P6)

Most participants updated the health diary on a daily basis, while a few updated it for several days at once. For some of the questions in the diary, a finer grading of choices was suggested.

### Lunches and Coffee Breaks

One diabetes participant missed the lunches from a conventional course; another one missed the coffee breaks that provide the chance to select a smaller group of peers to talk about personal matters not suitable for plenary discussion:

…during a break where you can chat with each other. About personal things, for example. Because there are after all many other things that go with diabetes, which might not be things you want to discuss in public. (P9)

One of the COPD participants suggested incorporating a small-talk session in the programme for more informal chat:

But I would have like a short break to chat, to put it like that, I mean, not just about the topic, but that we could talk about this and that. (P3)

### Conserving energy

An issue raised by one COPD participant was the need for conserving energy. If you are severely ill, it can be a challenge to prepare yourself for participation and to travel to the rehabilitation centre. By participating from home, you can conserve energy:

You have to get ready, get dressed, you're going to go out. It costs such an unbelievable amount of energy that you abandon the idea. It's so, you simply can't manage it. When you have it at home, of course you can grab your hairbrush, so that you look more or less OK (laughter) so, you can get up in the morning and take your medicine the way you always do, and get dressed, the things you need to be home on that day. You don't have to go anywhere. Whatever energy you have, you will still have it. (P3)

### Retention of participants

We had high participant retention and there were no dropouts from the study, but one person in the COPD group was unable to participate for two weeks due to a hospital admission. None of the participants reported that the duration of the home programmes was too long. Several of the COPD participants suggested that the home programme could be of longer duration.

## Discussion

### Principal results

Our findings indicate that the Internet-enabled home-group programmes for diabetes education and for pulmonary rehabilitation were acceptable to patients, and their perceptions of the form of delivery as well as the programme components were positive. In the home groups, interaction and dialogue were possible, despite less spontaneity and direct communication between peers compared to in-person group education. The group setting at home gives the participants a possibility to share experience and to learn from questions raised by others, questions and issues that they might not have thought of themselves, just as in conventional group education. The thematic educational videos were well accepted, despite lasting up to 40 minutes and not being of broadcast TV quality. The popularity of YouTube has also shown that quality aspects may be of less importance. Exercise training is an essential part of pulmonary rehabilitation [3,4]. At the outpatient rehabilitation centre, participants exercise together in a group, guided by the physiotherapist. Our findings indicate that the participants accepted group exercising organised in the same manner at home. Our findings also indicate that it may be possible to have groups at home without the participants meeting in person first, if not feasible for financial or practical reasons. The individual consultations were well perceived, but the need to discuss personal health issues varied among the participants.

Our study also gives some indications that it is possible to achieve a socially supportive environment in a group, with each person in their own home. The social aspect was emphasised by participants in the COPD group. Most of the COPD participants lived alone, while most diabetes participants did not. The diabetes participants were younger and they had fewer common group activities (e.g. no exercising together). All these aspects may have influenced the result. The social aspect may also be more important for people more bound to their homes and potentially more socially isolated, which is more likely to be the case for very severe COPD than for diabetes. Providing a socially supportive setting is a recognised part of pulmonary rehabilitation [3], while the rationale behind groups in diabetes self-management education is more of a practical and economic nature [8].

Overall, the results of our study indicate that group-based education and rehabilitation at home by means of an Internet-based TV-linked technology platform were well accepted.

### Generalisation and temporal aspects

The time elapsed since a study took place may influence the generalisation of the results due to factors such as changes in medical practice [22]. The technology might also become outdated.

Our trial prototype exploited the availability of powerful computers with a small form factor, and used them to make the living room TV interactive and “smart”. Our aim was a system that could also be used by people unfamiliar with computers. Even though the participants were satisfied with the video quality, the screen size of their CRT TV, and the dedicated computer, since then technological development has been in the direction of higher quality for less cost.

Today, a broad range of small powerful computers is available, capable of handling high-quality video with less bandwidth, which could be used as the Residential Patient Device. They are cheaper, smaller and less intrusive, lowering the technology cost of a home programme. Broadband penetration has also increased, with higher bandwidth at a lower cost. Modern flat-panel TVs have replaced CRT TVs in the consumer market, and offer large screen size of higher quality than their CRT predecessors offered. Recent years have also seen the introduction of smart TVs that are directly connected to the Internet. They have embedded computing power and they will support one of the application (apps) ecosystems (such as Google, Apple, Samsung and Microsoft). Connecting a microphone and a camera, if these are not embedded in the TV, will make it possible to run customised applications, supporting home programmes like the ones in the study.

The software system used in the prototype in the trials was mostly made by combining loosely coupled open-source software components in a web-based architecture. Today, one could orchestrate the same software system, using up-to-date open-source components, and provide the same service without much effort. The trial prototype exploited a simple browser-based user interface using HTML4; the new HTML5 standard will offer greater potential for a richer browser-based experience. This will however require some reimplementation, which also will be the case if the user interface is to be apps-based.

Given the fast technological development and expected penetration of smart TVs, the increase in broadband penetration and bandwidth, and support for an apps ecosystem, one can expect that in a few years’ time this infrastructure will be found in many homes as a platform for deploying easy-to-use health services to homes. For those without such infrastructure, however, both CRT TVs and flat-panel TVs can be used.

Developments in medical practice over time might require new programme design and content. However, there have not been any major changes in pulmonary rehabilitation statements or guidelines [3,4] or in diabetes education [7,8] since the study was performed that would affect the home programme design. Nor have there been significant changes in the practice at the outpatient clinic that ran the trials.

Technological development over time has worked in favour of these kinds of home-based programmes, while the prevalence of complex diseases such as COPD and diabetes continues to increase.

### Related work COPD

Limited research has been conducted on home-group based comprehensive pulmonary rehabilitation. The focus of home pulmonary rehabilitation has been on exercise training and physical activity in daily life and without group support; examples include [23-25].

Studies on live group exercising at home were performed by Taylor et al. [12,13]. They report on two trials with pulmonary rehabilitation at home with group exercise and advice sessions for COPD supervised by a physiotherapist. The trials were performed with groups of four and three patients respectively, using the patient’s TV connected to a PC at home. Their results also indicate that group exercising at home is feasible. They also found that the participants felt part of a group, and emphasised that structure was needed for turn taking in conversations. One weakness was the inability to support confidential one-to-one conversations with participants during the group sessions, which the physiotherapist solved by using the telephone for this purpose. Patient outcomes were comparable to those of conventional rehabilitation programmes.

For COPD, videoconferencing has been used to provide group education to users at remote centres [26], but not for group education at home.

### Related work diabetes

A review by Verhouven et al. [14] shows that videoconferencing has been used for individual diabetes case management at home in several studies, for example [27]. Diabetes self-management education using videoconferencing has been delivered at local clinics or community centres, both individually and in a group setting; examples include [28-30]. Ramadas et al. [31] found that web-based systems have been used for group discussion and peer support. We are, however, not aware of any related work on group diabetes self-management education using videoconferencing at home.

### Limitations

Due to the comprehensiveness and complexity of these home programmes, we have studied usability [10] and patient acceptability as a first step towards assessing their feasibility. The study results have provided valuable insight into usability and acceptability, but we have yet to assess the effect on health-related quality of life and exercise capacity, which are expected outcomes of outpatient group pulmonary rehabilitation. Nor have we assessed the effect of our home diabetes programme on blood glucose levels and knowledge about diabetes, or compared it with in-person group diabetes education.

A further limitation is the small number of participants, even though their characteristics varied regarding age, gender, family situation and computer skills. In addition, they were recruited through the same outpatient clinic that took part in the development and the running of the home programmes. This might bias the positive results and limit generalisation. Data on socioeconomic status were not collected for the participants. For people with COPD, distance is a barrier for uptake and completion of rehabilitation, regardless of the severity of the disease. In our study, the participants had very severe COPD, so the acceptability of the home programme to people with less severe COPD, who are potentially less homebound, is not known.

The healthcare personnel running the home programme had extensive experience in outpatient rehabilitation and education, which may have influenced user perception and therefore limit the applicability to treatment settings with less experienced personnel. A review by Deakin et al. [8] on group-based diabetes education shows that the outcome is not affected by which profession delivers the education, as long as the healthcare professionals are trained in delivery of education. The educational videos also featured the healthcare personnel delivering the group education, and it is not known how this may have influenced the results, and if new videos will be needed for each new treatment setting. The perceptions of the healthcare professionals on delivering the home programmes are not assessed in this paper.

We used a technology platform based on open source to keep costs low, but other financial aspects such as the total costs of offering the home-based programmes and possible reimbursement are not elaborated upon in this paper. We do not foresee a need for any major organisational changes in order to run these programmes regularly in clinical settings. However, organisational aspects need further investigation.

### Further work

Further work encompasses assessing acceptability for patients with less severe COPD, assessing acceptability to healthcare professionals, assessing patient outcomes, and further investigation of the possibility for a socially supportive environment in patient groups, with each person in their own home. Future work will include calculating costs of the home programmes compared with those of conventional programmes, and investigating any organisational changes needed.

## Conclusions

The Internet-enabled home-based group programmes for COPD and diabetes were generally well accepted by the participants. Our findings suggest that conventional programmes such as comprehensive multidisciplinary pulmonary rehabilitation and diabetes self-management education have the potential to be delivered in group settings at home. We also have some indications that it is possible to achieve a socially supportive environment in such home groups.

## Competing interests

The authors declare that they have no competing interests.

## Authors’ contribution

TMB, LVK, GØ, MR, AB, AH, TH, and TK contributed in the development of the home programmes. TMB and LKV contributed to the design of the technology platform and LKV to the development. GØ made the videos. MR, AB, AH and TH contributed to patient recruitment and teaching in the home programmes. GØ performed the interviews. GØ, TMB and MB structured the interview material, and GØ, TMB, MB, LKV and EJ analysed the material. TMB, LKV and EJ prepared the manuscript, which has been reviewed by the other authors. All authors have approved the final manuscript.

## Pre-publication history

The pre-publication history for this paper can be accessed here:

http://www.biomedcentral.com/1472-6947/13/33/prepub
